# Multicenter retrospective observational study on the clinical effectiveness of butyrate-producing *Clostridium butyricum* containing probiotics in patients with COVID-19

**DOI:** 10.1080/21505594.2026.2673650

**Published:** 2026-05-13

**Authors:** Yoshihiko Morikawa, Hideo Kato, Takumi Umemura, Jun Hirai, Yuichi Shibata, Mao Hagihara, Nobuhiro Asai, Hiroshige Mikamo, Takuya Iwamoto

**Affiliations:** a Department of Pharmacy, Mie University Hospital, Mie, Japan; b Department of Clinical Infectious Diseases, Aichi Medical University, Nagakute, Japan; c Department of Pharmacy, Tosei General Hospital, Seto, Japan; d Division of Infection Control and Prevention, Nippon Medical School Chiba Hokusoh Hospital, Chiba, Japan; e Department of Molecular Epidemiology and Biomedical Sciences, Aichi Medical University, Nagakute, Japan

**Keywords:** COVID-19, probiotics, butyrate-producing bacteria, secondary bacterial pneumonia

## Abstract

Butyrate-producing bacteria, which are components of the gut microbiome, activate host defense mechanisms against several types of infections, including respiratory viral infections. However, the clinical effectiveness of butyrate-producing *Clostridium butyricum* (CB)-containing probiotics in patients with coronavirus disease 2019 (COVID-19) remains unclear. We investigated the in-hospital mortality, period of mechanical ventilation, and incidence of secondary bacterial pneumonia in patients with COVID-19 from 2020 to 2021. The patients were divided into the probiotic (27) and non-probiotic (256) groups. The two groups did not show a significant difference in the SOFA scores (probiotic vs. non-probiotic, 2.1 ± 2.3 vs. 2.1 ± 2.9). Additionally, all patients received antiviral agents to treat COVID-19; however, the two groups did not show significant difference in their distribution. However, patients receiving CB preparations showed the shorter periods of mechanical ventilation (1.1 ± 2.5 days vs. 3.9 ± 9.4 days). Although not statistically significant, they also showed lower incidence of secondary bacterial pneumonia (7.4% vs. 15.6%) and the lower in-hospital mortality (3.7% vs. 15.2%) compared to the non-probiotic group. This retrospective clinical study revealed that the administrations of CB preparations might attenuate clinical symptoms related to COVID-19 and improve mortality. However, further clinical and basic studies are required to validate our findings.

## Introduction

Severe acute respiratory syndrome coronavirus 2 (SARS-CoV-2), which is responsible for the coronavirus disease 2019 (COVID-19), has resulted in over 770 million infections and 7 million deaths worldwide [1]. Antiviral agents, immunosuppressants, and modulators are recommended as treatments, and vaccines are recognized as a significant option to prevent the worsening of viral infection [2]. Despite the advent of highly effective vaccines against SARS-CoV-2, the overall vaccination rate remains suboptimal [3]. Moreover, the effectiveness of vaccines in enhancing immunity against COVID-19 increases over time [4]. Therefore, a safe, low-cost, and rapidly implementable alternative for the treatment of COVID-19 is necessary.

Probiotics have attracted significant attention for their ability to affect the host immune system [5]. For example, orally administered probiotics may lead to the upregulation of interferons, which enhance the bactericidal functions of macrophages [6]. Stimulation of the type 1 immune response of T helper cells increases the synthesis of secretory Immunoglobulin A leading to the clearance of influenza virus in the lungs [7]. Furthermore, probiotics can enhance antiviral effects in patients with viral infections when co-administered with various antiviral agents [8], and probiotics have prophylactic effects against respiratory infections in healthy adults and children [9]. Therefore, probiotics may play an important role in preventing and managing COVID-19.

Butyrate, a short-chain fatty acid and major metabolite produced by the gut microbiota, has been reported to regulate several host defense mechanisms against infections, including the modulation of gene expression related to antiviral pathways [10–12]. It has also been reported that the abundance of butyrate-producing bacteria is significantly reduced in COVID-19 patients [13], and that changes in the intestinal flora affect the severity of COVID-19 [14]. Hence, the intake of probiotics containing butyrate-producing bacteria, such as CB, may be particularly effective in the treatment of COVID-19. While CB includes various strains, some of which possess toxin-producing genes [15], nonpathogenic strains with well-established safety – such as the MIYAIRI 588 strain (CBM 588) – have been widely used as probiotics in Japan for several decades. Today, this strain is commercially available as a probiotic for the treatment of antibiotic-associated diarrhea and other gastrointestinal disorders. The large-scale production and distribution of probiotics containing CB are already well established, making it a practical and accessible candidate for adjunctive treatment of COVID-19.

However, the clinical effects of CB preparations as a concomitant medication with existing therapies, such as antiviral agents for COVID-19 have remained unclear. Hence, this study aimed to evaluate clinical effectiveness of two CB preparations commercially available in Japan – MIYA-BM® and BIO-THREE®—for the treatment of COVID-19 in patients receiving antiviral agents. These two probiotic formulations are commercially available in Japan and have been widely used for many years, with established safety profiles. For this reason, they were selected as the focus of the present study.

## Patients and methods

### Patient characteristics

We retrospectively reviewed the medical records of patients hospitalized with COVID-19 between August 2020 and June 2021 at Mie University Hospital, Tosei General Hospital, and Aichi Medical University Hospital who were treated with antiviral agents. The following patients were excluded from the study: those who did not receive any antiviral agents, those transferred to other hospitals for COVID-19 treatment, and those who received probiotics other than CB preparations. We defined patients who received CB preparations as the CB preparation group and those who did not receive probiotics as the non-probiotic group. For a sub-analysis, patients who received CB preparations were divided into patients who received MIYA-BM® (containing *C. butyricum*) or BIO-THREE® (containing *C. butyricum*, *Enterococcus faecium*, and *Bacillus subtilis*). This study was conducted in accordance with the ethical principles of the Declaration of Helsinki and approved by the Ethics Committee of Mie University Hospital (No. H2022-179).

### Data collection

Patient background information, including sex, age, body weight, body mass index, presence of underlying conditions, sequential organ failure assessment (SOFA) score, and laboratory data on admission were collected from medical records. Diabetes, hypertension, and cardiovascular diseases, which contribute to COVID-19 exacerbation, have also been investigated [16]. In addition, information on the CB preparations including product name, daily dosage, dosing frequency, and duration of administration was also collected retrospectively from the medical records.

### Outcomes

The primary outcome was in-hospital mortality rate. Secondary outcomes were the duration of mechanical ventilation and the presence of secondary bacterial pneumonia. Secondary bacterial pneumonia was the presence of bacterial growth in sputum cultures collected from patients during hospitalization, with bacteria considered the causative pathogen of the pneumonia.

### Statistical analysis

All statistical analyses were performed using JMP Pro 16 software (SAS Institute, Cary, NC, USA). Statistical significance was set at *p* < 0.05, unless otherwise indicated. Normally distributed continuous data were presented as the mean ± standard deviation and were compared using analysis of variance. Non-normally distributed continuous data are presented as median (interquartile range). Statistical significance was evaluated using an unpaired *t*-test for continuous data. Categorical data are presented as numbers (%) and were compared using the chi-square test.

## Results

### Patients

Overall, 283 patients met the inclusion criteria during the study period (Mie University Hospital, *n* = 39; Tosei General Hospital, *n* = 111; Aichi Medical University Hospital, *n* = 133). Of these, 27 patients (9.5%) received CB preparations and 256 patients (90.5%) did not. Favipiravir was administered to 76 patients (26.9%), remdesivir to 154 patients (54.4%), casirivimab/imdevimab to 3 patients (1.1%), a combination of favipiravir and remdesivir to 41 patients (14.5%), a combination of remdesivir and casirivimab/imdevimab to 8 patients (2.8%), and a combination of remdesivir and sotrovimab to 1 patient (0.3%). There were no significant differences in the distribution of antiviral agents between the MIYA-BM® and BIO-THREE® groups.

Among the 27 patients who received CB preparations, 17 received MIYA-BM® and 10 received BIO-THREE®. In the MIYA-BM® group (*n* = 17), seven patients received the tablet formulation, which contains 20 mg of CB per tablet, and ten patients received the powder formulation, containing 40 mg of CB per gram. The administration of MIYA-BM® was initiated at a mean of 4 days after hospital admission (range: 1–7 days), with a mean duration of 7 days (range: 2 –41 days). In the BIO-THREE® group (*n* = 10), eight patients received the tablet formulation, containing 10 mg of CB per tablet, and two patients received the powder formulation, containing 50 mg of CB per gram. The administration of BIO-THREE® was initiated at a mean of 2.5 days after hospital admission (range: 1–6 days), with a mean duration of 10 days (range: 5–49 days). Importantly, there were no statistically significant differences between the MIYA-BM® and BIO-THREE® groups in terms of the timing of initiation (3.5 ± 2.0 days vs. 2.9 ± 1.7 days, *p* = 0.4422) or the duration of administration (10.4 ± 9.5 days vs. 16.6 ± 15.0 days, *p* = 0.1814). The dosage for all patients complied with the approved dosage specified in the package insert in Japan. In patients treated with MIYA-BM®, four patients (23.5%) received favipiravir, 10 patients (58.8%) received remdesivir, one patient (5.9%) received casirivimab/imdevimab, and two patients (11.8%) received a combination of favipiravir and remdesivir. Among patients treated with BIO-THREE®, four patients (40.0%) received favipiravir, and six patients (60.0%) received remdesivir.

The demographic and clinical characteristics of the patients are shown in Table 1. The median age and weight (with ranges) for the CB preparations and non-probiotic groups were as follows: 64 years (22–94 years) and 72.5 kg (33.7–92.6 kg) for the CB preparations group, and 62 years (24–91 years) and 66.0 kg (35.4–197.2 kg) for non-probiotic group. A larger proportion of patients were male (CB preparation group, *n* = 20, 74.1%; non-probiotic group, *n* = 175, 68.4%). There was no significant difference in the SOFA scores at admission between the CB preparation and non-probiotic groups (CB preparation group vs. non-probiotic group, 1 [0–7] vs 1 [0–14], *p* = 0.5048). Moreover, there were no significant differences in laboratory data, underlying diseases, or antiviral agents between the patients who received CB preparations and those who did not.

**Table 1.** Clinical characteristics of the patients.

	CB preparations	Non-probiotic	*p*-value
Sex (male/female)	20/7	175/81	0.5418
Age (years)	64 [22–94]	62.0 [24–91]	0.4933
Body weight (kg)	72.5 [33.7–92.6]	66.0 [35.4–197.2]	0.9704
Body Mass Index (kg/m^2^)	24.6 [13.7–32.8]	24.3 [14.9–69.0]	0.8957
eGFR (mL/min/1.73 m^2^)	69.8 ± 56.6	71.4 ± 32.4	0.1523
BUN (mg/dL)	24.3 ± 18.6	18.9 ± 12.4	0.2373
AST (IU/L)	48.4 ± 30.7	56.2 ± 47.9	0.4567
ALT (IU/L)	40.7 ± 33.2	42.5 ± 38.0	0.8781
WBC (10^3^/μL)	6.8 ± 2.9	7.0 ± 3.7	0.9527
CRP (mg/dL)	7.9 ± 7.0	8.1 ± 6.8	0.8175
SOFA score	1 [0–7]	1 [0–14]	0.5048
Underlying conditions (%, n)			
Diabetes	33.3 (9/27)	30.5 (78/256)	0.7590
Hypertension	25.9 (7/27)	30.5 (78/256)	0.6243
Cardiovascular Disorders	7.4 (2/27)	8.6 (22/256)	0.8333

Age, Body weight, Body Mass Index and SOFA scores are shown as medians [minimum to maximum]. eGFR, BUN, AST, ALT, WBC, CRP are shown as mean ± standard deviation. CB preparation group: patients receiving CB preparations (*n* = 27); non-probiotic group: patients not receiving probiotics (*n* = 256).

eGFR, estimated glomerular filtration rate; BUN, blood urea nitrogen; AST, aspartate aminotransferase; ALT, alanine aminotransferase; WBC, white blood cells; CRP, C-reactive protein; SOFA, Sequential Organ Failure Assessment.

### Clinical effectiveness

Comparisons of in-hospital mortality, period of mechanical ventilation, and incidence of pneumonia between the CB preparations and non-probiotic groups are shown in Table 2. The administration of CB preparations to patients with COVID-19 reduced their in-hospital mortality; however, the difference was not significant (CB preparation group vs. non-probiotic group, 3.7% vs. 15.2%, *p* = 0.1019). Compared to the non-probiotic group, patients in the CB preparations group had significantly shorter duration of mechanical ventilation (1.1 ± 2.5 days vs 3.9 ± 9.4 days, *p* = 0.0010) and reduced incidence of secondary bacterial pneumonia (7.4% vs 15.6%, *p* = 0.2533).

**Table 2.** Comparison of clinical effectiveness between CB preparations and non-probiotic groups.

	CB preparations	Non-probiotic	*p*-value
Secondary bacterial pneumonia (%, n)	2(7.4)	40(15.6)	0.2533
Duration of mechanical ventilation (days)	1.1 ± 2.5	3.9 ± 9.4	0.0010
In-hospital mortality rate (%)	1(3.7)	39(15.2)	0.1019

The duration of mechanical ventilation is shown as mean ± standard deviation. CB preparation group: patients receiving CB preparations (*n* = 27); non-probiotic group: patients not receiving probiotics (*n* = 256).

### Clinical effectiveness in patients receiving MIYA-BM®

The comparison of patients’ backgrounds and clinical effectiveness between the MIYA-BM® and non-probiotic groups are shown in Tables 3 and 4. MIYA-BM® administration decreased the in-hospital mortality, shortened the period of mechanical ventilation, and decreased the incidence of secondary bacterial pneumonia, but did not show a significant difference (mortality, 5.9% vs 15.2%, *p* = 0.2910; duration of mechanical ventilation, 1.6 ± 3.2 vs 3.9 ± 9.4 days, *p* = 0.6386; incidence of secondary bacterial pneumonia, 11.8% vs 15.6%, *p* = 0.6692).

**Table 3.** Clinical characteristics of MIYA-BM® group and non-probiotic group.

	MIYA-BM®	Non-probiotic	*p*-value
Sex (male/female)	14/3	175/81	0.2261
Age (years)	64 [32–94]	62.0 [24–91]	0.5776
Body weight (kg)	75.5 [33.7–92.6]	66.0 [35.4–197.2]	0.2256
Body Mass Index (kg/m^2^)	26.7 [13.7–32.8]	24.3 [14.9–69.0]	0.2885
eGFR (mL/min/1.73 m^2^)	61.2 ± 27.4	71.4 ± 32.4	0.1613
BUN (mg/dL)	25.4 ± 19.4	18.9 ± 12.4	0.2829
AST (IU/L)	49.8 ± 33.2	56.2 ± 47.9	0.5951
ALT (IU/L)	46.9 ± 37.6	42.5 ± 38.0	0.4881
WBC (10^3^/μL)	8.0 ± 3.1	7.0 ± 3.7	0.0878
CRP (mg/dL)	9.4 ± 8.3	8.1 ± 6.8	0.6638
SOFA score	1 [0–7]	1 [0–14]	0.7617
Underlying conditions (%, n)			
Diabetes	35.3 (6/17)	30.5 (78/256)	0.6764
Hypertension	35.3 (6/17)	30.5 (78/256)	0.6764
Cardiovascular Disorders	11.8 (2/17)	8.60 (22/256)	0.6548

Age, Body weight, Body Mass Index and SOFA scores are shown as medians [minimum to maximum]. eGFR, BUN, AST, ALT, WBC and CRP are shown as mean ± standard deviation. MIYA-BM® group, patients receiving MIYA-BM® (*n* = 17); Non-probiotic group, patients not receiving probiotics (*n* = 256).

eGFR, estimated glomerular filtration rate; BUN, blood urea nitrogen;AST, aspartate aminotransferase; ALT, alanine aminotransferase; WBC, whiteblood cells; CRP, C-reactive protein; SOFA, SequentialOrgan Failure Assessment.

**Table 4.** Comparison of clinical effectiveness between the MIYA-BM® and non-probiotic groups.

	MIYA-BM®	Non-probiotic	*p*-value
Secondary bacterial pneumonia (%, n)	2(11.8)	40(15.6)	0.6692
Duration of mechanical ventilation (days)	1.6 ± 3.2	3.9 ± 9.4	0.6386
In-hospital mortality (%)	1(5.9)	39(15.2)	0.2910

The duration of mechanical ventilation is shown as mean ± standard deviation. MIYA-BM® group, patients receiving MIYA-BM® (*n* = 17); Non-probiotic group, patients not receiving probiotics (*n* = 256).

### Clinical effectiveness in patients receiving BIO-THREE®

The comparison of patients’ backgrounds and clinical effectiveness between the BIO-THREE® and non-probiotic groups are shown in Tables 5 and 6. Similar to the analysis between the patients receiving MIYA-BM® and non-probiotic groups, BIO-THREE® administration decreased the in-hospital mortality, shortened the period of mechanical ventilation, and decreased the incidence of secondary bacterial pneumonia, but did not show a significant difference (mortality, 0% vs 15.6%, *p* = 0.1751; duration of mechanical ventilation, 0.4 ± 1.1 vs 3.9 ± 9.4 days, *p* = 0.3343; incidence of secondary bacterial pneumonia, 0% vs 15.6%, *p* = 0.1751). No patients died, and none of the patients treated with BIO-THREE® developed secondary bacterial pneumonia.

**Table 5.** Clinical characteristics of BIO-THREE® group and non-probiotic group.

	BIO-THREE®	Non-probiotic	*p*-value
Sex (male/female)	6/4	175/81	0.5781
Age (years)	64 [22–84]	62.0 [24–91]	0.6705
Body weight (kg)	54.8 [48.0–76.0]	66.0 [35.4–197.2]	0.0941
Body Mass Index (kg/m^2^)	22.4 [19.0–26.3]	24.3 [14.9–69.0]	0.0784
eGFR (mL/min/1.73 m^2^)	84.4 ± 86.8	71.4 ± 32.4	0.5645
BUN (mg/dL)	22.5 ± 18.1	18.9 ± 12.4	0.5589
AST (IU/L)	46.0 ± 27.6	56.2 ± 47.9	0.5758
ALT (IU/L)	30.2 ± 21.8	42.5 ± 38.0	0.2398
WBC (10^3^/μL)	4.8 ± 1.1	7.0 ± 3.7	0.0185
CRP (mg/dL)	5.4 ± 3.5	8.1 ± 6.8	0.3344
SOFA score	2 [0–5]	1 [0–14]	0.4649
Underlying conditions (%, n)			
Diabetes	30.0 (3/10)	30.5 (78/256)	0.9748
Hypertension	10.0 (1/10)	30.5 (78/256)	0.1646
Cardiovascular Disorders	0 (0/10)	8.60 (22/256)	0.3331

Age, Body weight, Body Mass Index and SOFA scores are shown as medians [minimum to maximum]. eGFR, BUN, AST, ALT, WBC and CRP are shown as mean ± standard deviation. BIO-THREE® group, patients receiving BIO-THREE® (*n* = 10); Non-probiotic group, patients not receiving probiotics (*n* = 256).

eGFR, estimated glomerular filtration rate; BUN, blood urea nitrogen; AST, aspartate aminotransferase; ALT, alanine aminotransferase; WBC, white blood cells; CRP, C-reactive protein; SOFA, Sequential Organ Failure Assessment.

**Table 6.** Comparison of clinical effectiveness between the BIO-THREE® and non-probiotic groups.

	BIO-THREE®	Non-probiotic	*p*-value
Secondary bacterial pneumonia, n (%)	0(0)	40(15.6)	0.1751
Duration of mechanical ventilation (days)	0.4 ± 1.1	3.9 ± 9.4	0.3343
In-hospital mortality (%)	0(0)	39(15.2)	0.1815

The duration of mechanical ventilation is shown as mean ± standard deviation. BIO-THREE® group, patients receiving BIO-THREE® (*n* = 10); Non-probiotic group, patients not receiving probiotics (*n* = 256).

## Discussion

Patients who received CB preparations showed lower in-hospital mortality, a shorter duration of mechanical ventilation, and a reduced incidence of secondary bacterial pneumonia compared with those who did not receive any probiotics. Although the sample size was limited, these findings suggest that CB preparations may contribute to improved clinical outcomes in patients with COVID-19.

Gut microbiota influence host physiological functions through microbial-derived metabolites [17]. Short-chain fatty acids (SCFAs) activate dendritic cell functions and enhance immune responses against pathogens [18]. Specifically, butyrate induces the differentiation of regulatory T cells (Tregs) and maintains immune homeostasis in the gut [19]. As the concept of the “gut – lung axis,” wherein gut microbiota influences immune responses in the lungs, has been proposed [20], CB preparation may regulate immune responses in the lungs by restoring gut microbiota. Previous studies have shown that modulation of the gut microbiota, particularly through enrichment of SCFA-producing bacteria, can attenuate secondary bacterial infections following viral pneumonia. Sencio V et al. [21] and Verstraeten S et al. [22] demonstrated in murine models that enhancing SCFA levels via dietary fiber or probiotics improved host resistance to superimposed bacterial pneumonia, suggesting a potential mechanism applicable to COVID-19. While definitive evidence is still lacking, these findings support the hypothesis that probiotics, particularly those promoting SCFA production, may have a beneficial adjunctive role in COVID-19 management. Our results revealed that CB preparation reduced the severity of pneumonia following the onset of COVID-19. These findings suggest that CB preparations can reduce the severity of pneumonia by enhancing the immune response.

We also found that the duration of mechanical ventilation was shorter in the group treated with CB preparations than in the non-probiotic group. These findings suggest that CB preparations may be effective for treating COVID-19. Patients who received transplanted allogeneic hematopoietic stem cells with a higher relative abundance of butyrate-producing bacteria in the gut microbiome have been associated with enhanced resistance to respiratory viral infections [23]. Therefore, butyrate-producing bacteria in the gut microbiota may reduce the risk of viral infections in the lower respiratory tract viral infections [24]. Furthermore, a recent *in vivo* study revealed a detailed mechanism underlying the viral infection-resistance effects of orally administered CB preparations. CB-induced ω-3 fatty acid 18-hydroxy eicosapentaenoic acid (18-HEPE) has enhanced interferon (IFN)-λ production in pulmonary epithelial cells through the activation of the G protein-coupled receptor (GPR)120 and IFN regulatory factor (IRF)-1/-7 [25]. Therefore, our findings may be due to antiviral effects via the gut – lung axis mechanism. However, there was no significant difference in-hospital mortality between the CB preparation and non-probiotic groups, whereas it was lower in the CB preparation group than in the non-probiotic group. During COVID-19, worsening pulmonary symptoms leading to acute respiratory distress syndrome requiring mechanical ventilation have been reported to increase mortality [26]. In this study, the short duration of mechanical ventilation in the CB preparation group may have contributed to the reduction in mortality. Thus, CB preparation may show clinical efficacy by avoiding the aggravation of pulmonary symptoms in patients with COVID-19.

Antiviral effects, including immune regulatory effects, vary depending on the bacterial strain, including probiotics [27]. In our study, no cases of secondary bacterial pneumonia or death were observed in patients who received the BIO-THREE®, which includes butyrate-producing *C. butyricum* and lactic acid bacteria and saccharifying bacteria (*S. faecalis* and *B. mesentericus*). Saccharifying bacteria are microorganisms that produce enzymes capable of breaking down complex carbohydrates into simpler sugars, such as glucose, which can then be fermented by other gut microbes to produce SCFAs like butyrate. This metabolic cooperation may enhance butyrate production and improve gut immune function. This suggests that the combined use of multiple types of bacteria may be more effective than the use of butyrate-producing bacteria alone. Butyrate-producing bacteria play an important role in enhancing resistance to influenza viral infection [24], and butyrate production requires lactic acid [28]. Lactic acid-producing bacteria were present in BIO-THREE®. Moreover, saccharifying producing bacteria, contained in the BIO-THREE®, promotes growth of butyrate-producing bacteria [29]. Therefore, these synergistic actions would indicate the superior clinical effectiveness in the BIO-THREE® with multiple bacteria including lactic acid-producing bacteria and saccharifying-producing bacteria than CB preparation with single bacteria for COVID-19 patients.

This study had some limitations. First, it employed a retrospective observational design, which introduces the possibility of selection and information biases. The decision to administer CB preparations was not randomized and may have been influenced by unmeasured clinical factors, limiting our ability to infer causality. Second, patients with mild disease who did not require hospitalization were excluded. Therefore, whether CB preparations should be used in combination with antiviral agents depending on the severity of COVID-19 is a subject for future studies. Third, although the dosage form, dose, and duration of CB preparations were extracted from medical records, these were not standardized across patients and may have acted as confounding variables. However, there were no statistically significant differences between the MIYA-BM® and BIO-THREE® groups in terms of the timing of initiation or duration of administration, suggesting that their influence as confounding factors may have been limited. Nonetheless, the lack of a unified treatment protocol means that residual confounding cannot be entirely ruled out. Fourth, the sample size of patients who received CB preparations was relatively small. Therefore, further studies with larger cohorts are necessary to validate these findings. Fifth, while we adjusted for some baseline characteristics, other important factors such as prior health status and concomitant medications may have influenced outcomes but were not fully controlled. Sixth, we were unable to examine the impact of SARS-CoV-2 variants. SARS-CoV-2 has evolved genetically rapidly, resulting in multiple subtypes with intricate mutations [30]. Transmissibility and severity vary by variant [31]. This study was unable to determine the specific variants of the virus in patients based on clinical data. Further research that considers the influence of these variants is necessary. Seventh, this study did not include microbiome profiling, quantitative analysis of *C.butyricum* in fecal samples, or measurement of short-chain fatty acids such as butyrate. Therefore, we could not evaluate potential correlations between microbiota composition, butyrate levels, and clinical outcomes. Further studies addressing these points are warranted. Finally, this study did not include detailed microbiological analyses of secondary bacterial infections. Therefore, the specific bacterial species responsible for these infections could not be identified, which limits our ability to evaluate the potential association between CB administration and the suppression of particular pathogens.

In conclusion, this study revealed that butyrate-producing *C. butyricum* containing preparations reduced the risk of COVID-19-related severe dysfunction and improved COVID-19 related symptoms. Specifically, CB preparations containing *S. faecalis* and *B. mesentericus* enhanced the clinical efficacy against COVID-19. However, further clinical and basic studies are required to validate our findings.

## Data Availability

The datasets generated and/or analyzed during the current study are not publicly available due to patients’ privacy but are available from the corresponding author on reasonable request.
